# Amino Acids 563–566 of the Na^+^/H^+^ Exchanger Isoform 1 C-Terminal Cytosolic Tail Prevent Protein Degradation and Stabilize Protein Expression and Activity

**DOI:** 10.3390/ijms21051737

**Published:** 2020-03-03

**Authors:** Xiuju Li, Debajyoti Dutta, Martin Jung, Richard Zimmermann, Larry Fliegel

**Affiliations:** 1 Department of Biochemistry, University Alberta, Edmonton, AB T6G 2H7, Canada; xjli@ualberta.ca (X.L.); debajyoti.47@gmail.com (D.D.); 2 Department of Biotechnology, Thapar Institute of Engineering and Technology Patiala, Patiala 147004, Punjab, India; 3 Medical Biochemistry and Molecular Biology, Saarland University, 66421 Homburg, Germany; Martin.Jung@uks.eu (M.J.); richard.zimmermann@uks.eu (R.Z.)

**Keywords:** membrane protein, Na^+^/H^+^ exchanger, pH regulation, protein degradation, protein stability

## Abstract

Isoform one of the mammalian Na^+^/H^+^ exchanger is a plasma membrane protein that is ubiquitously present in humans. It regulates intracellular pH through the removal of one intracellular proton in exchange for a single extracellular sodium. It consists of a 500 amino acid membrane domain plus a 315 amino acid, C-terminal tail. We examined amino acids of the C-terminal tail that are important in the targeting and activity of the protein. A previous study demonstrated that stop codon polymorphisms can result in decreased activity, expression, targeting and enhanced protein degradation. Here, we determine elements that are critical in these anomalies. A series of progressive deletions of the C-terminal tail demonstrated a progressive decrease in activity and targeting, though these remained until a final drop off with the deletion of amino acids 563–566. The deletion of the ^562^LIAGERS^568^ sequence or the alteration to the ^562^LAAAARS^568^ sequence caused the decreased protein expression, aberrant targeting, reduced activity and enhanced degradation of the Na^+^/H^+^ exchanger (NHE1) protein. The ^562^LIAGERS^568^ sequence bound to other regions of the C-terminal cytosolic domain. We suggest this region is necessary for the activity, targeting, stability, and expression of the NHE1 protein. The results define a new sequence that is important in maintenance of NHE1 protein levels and activity.

## 1. Introduction

The mammalian Na^+^/H^+^ exchanger (NHE1, isoform 1) is a ubiquitously expressed membrane protein of mammalian cells. It removes one intracellular proton in exchange for a single extracellular sodium ion. NHE1 has several important physiological functions. It maintains intracellular pH (pH_i_) while protecting cells from acidification as a result of metabolism. It also regulates cell volume in response to osmotic challenge. There are ten isoforms of NHE that are different gene products [1,2,3,4,5,6]. In human pathology, NHE1 is critical in several diseases, including myocardial heart hypertrophy and ischemia reperfusion damage [7]. NHE1 is also important in breast cancer, where it acts as a trigger for metastasis [8,9].

NHE1 consists of two domains: a membrane domain that transports ions and a C-terminal cytosolic regulatory domain. The N-terminal membrane domain is comprised of 500 amino acids, and the C-terminal cytosolic regulatory domain is 315 amino acids long and functions to regulate the membrane domain [10]. Genetic mutations in NHE1 have been previously documented. A severe mutation in the human NHE1 gene *SLC9A1* causes ataxia and deafness in a disease called Lichtenstein–Knorr syndrome [11]. Another genetic mutation in the transmembrane region of NHE1 also abolishes activity [12]. We [13] recently examined the effects of a number of mutations that result in stop codon polymorphisms in the NHE1 protein; an NHE1 mutant with deleted C-terminal 80 amino acids had only minor defects. Surprisingly, truncating the NHE1 protein much more at amino acid 543 resulted in greatly reduced NHE1 activity. This was caused by a reduced expression and surface localization. The smaller protein showed an enhanced degradation, though RNA stability was unchanged.

In the present study, we examine the proximal region of the NHE1 tail in more detail in order to understand the regions and factors that are involved in mediating the decreased activity, targeting, and enhanced degradation of the protein. Shortening the NHE1 C-terminal cytosolic tail results in a gradual decrease in the expression and activity of the protein. Notably, we discover that amino acids 563–566 are critical for stability of the protein. Their mutation or shortening the cytosolic tail past these amino acids causes the enhanced degradation and decreased activity of the NHE1 protein. The results characterize a novel sequence that is important in maintenance of NHE1 protein levels and activity.

## 2. Results

### 2.1. Construction and Characterization of NHE Mutants

**Figure 1:**
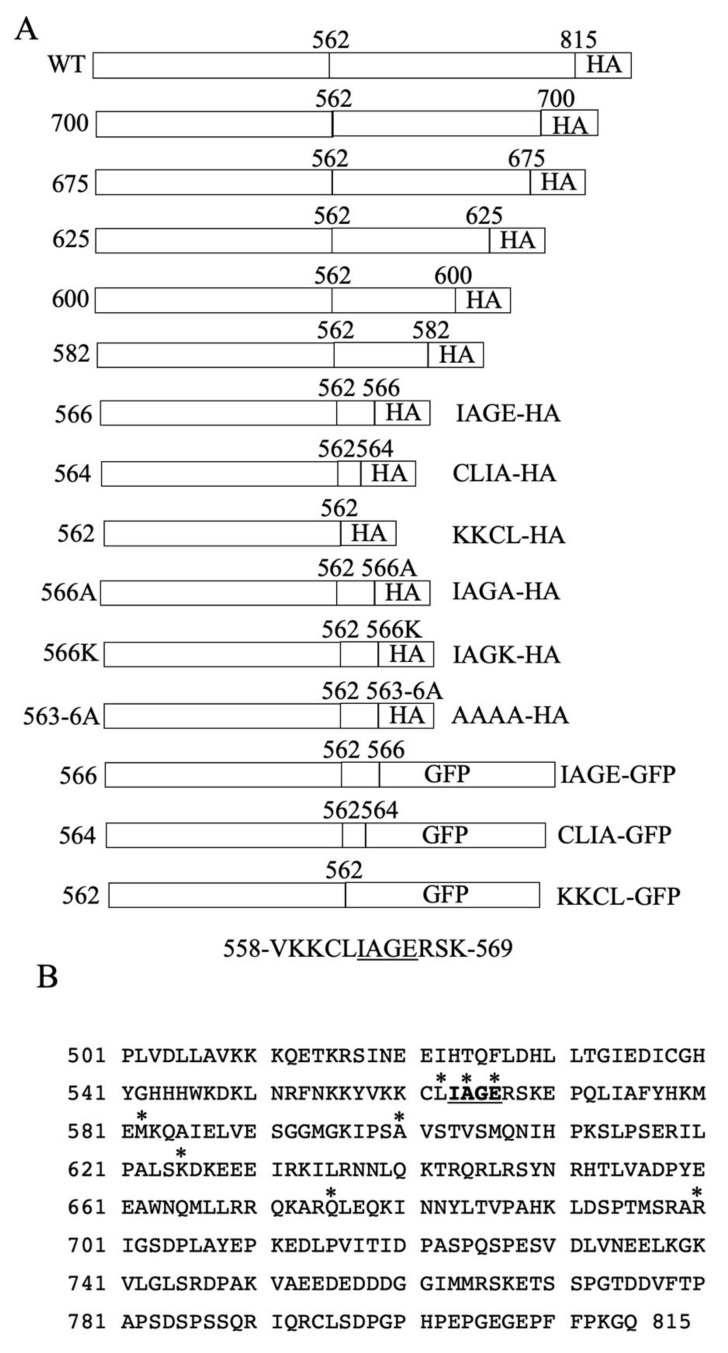
Na^+^/H^+^ exchanger (NHE1) sequence and constructs. (**A**) Schematic diagram of NHE1 constructs shortened at the carboxyl terminal region. All proteins were expressed from the initiator methionine of NHE1. Wild type (WT), full length NHE1 with an HA (hemagglutinin) tag. The number of the last amino acid of the C-terminus of NHE1 is indicated. For shorter constructs, the last four amino acids prior to the HA (or GFP) tag are shown. GFP indicates a green fluorescent protein tag. The sequence of amino acids 558–569 of NHE1 is shown below. (**B**) Amino acid sequence of the human NHE1 cytosolic regulatory domain, amino acids 501–815. Amino acids 563–566 are underlined and bold. * above an amino acid indicates the position where the protein’s C-terminal was terminated in order to form that amino acid.

**Figure 2:**
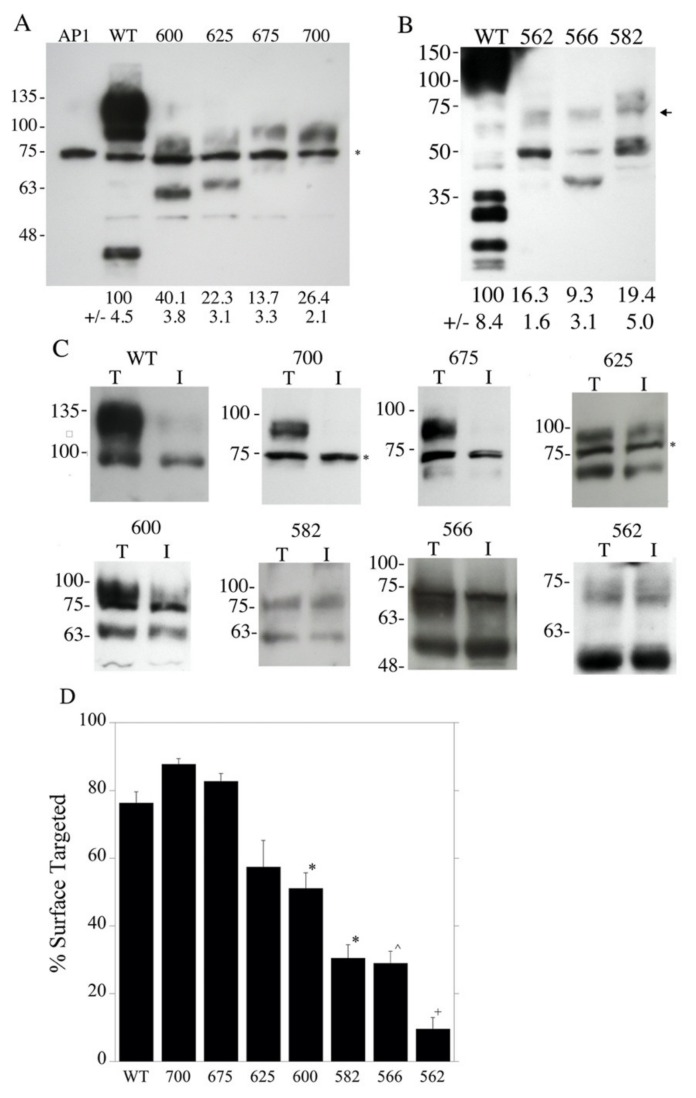
Expression and targeting of wild type NHE1 and mutants NHE-562, NHE-566, NHE-582, NHE-600, NHE-625, NHE-675 and NHE-700. (**A**) Western blot of whole cell lysates of stably transfected AP1 cells containing the indicated plasmids NHE-600, NHE-625, NHE-675 and NHE-700. Asterisk indicates a non-specific immunoreactive band that was present in all cell lysates. WT, wild type. AP1, mock transfected AP1 cells. The level of expression, relative to the wild type, is indicated below each lane. * indicates non-specific immunoreactive protein. (**B**) Analysis of mutant expression as in “A” with mutants NHE-562, NHE-566 and NHE-582. Exposure time was increased to allow for the visualization of the mutant expression. Arrow indicates the approximate position of the shortened NHE1 proteins. (**C**) Surface localization of the wild type (WT) and mutant NHE1 proteins in AP1 cells. The results are mean ± the S.E, *n* = at least 3 determinations. After external cell surface biotinylation, equal amounts of total cell lysates (T) and unbound intracellular lysates (I) were examined by Western blotting with anti-HA antibody to identify the NHE1 protein. WT and mutant cell lines (NHE-562, NHE-566, NHE-582, NHE-600, NHE-625, NHE-675 and NHE-700 are indicated). Lysates were from cell lines stably expressing wild type NHE1 and mutant NHE1 proteins, respectively. * indicates non-specific immunoreactive protein. (**D**) Summary of surface localization of WT and mutant NHE1 proteins in AP1 cells (as in “C”). * indicates values that were significantly different from the control values at *p* < 0.05. ^ indicates significantly values that were different from control values at *p* < 0.01. + indicates significantly different from NHE-566 at *p* < 0.05.

A previous study [13] shortened the carboxyl terminal tail of NHE1 in a manner that was equivalent to reported human genetic polymorphisms. This resulted in the defective activity, expression and targeting of the protein. To investigate this further, a series of mutants were made that reduced the length of the carboxyl terminal end of NHE1 from amino acid 815 to amino acid 562 (Figure 1). The first series of examined mutants stopped at amino acids 700, 675, 625, 600, 582, 566 and 562. Figure 2A illustrates the wild type protein and the mutants with the C-terminal tail terminating at amino acids 700–562. The wild type NHE1 protein was present as two major bands that represent a fully glycosylated protein and a partial or de-glycosylated protein [14]. The mutant proteins were reduced in expression levels compared to the wild type protein. The NHE1 proteins were expressed as two forms—one with a higher apparent molecular weight that was possibly glycosylated and the other as a reduced molecular weight immunoreactive species (though in some cases the reduced molecular weight form may have been obscured by a non-specifically cross-reacting protein). The shortened mutant forms of the NHE1 protein terminating at amino acids 625 and 600 appeared to have relatively more reduced molecular weight, immunoreactive (presumably un-glycosylated) species, while the wild type, NHE-675, and NHE-700 proteins had a higher proportion of higher apparent molecular weight immunoreactive NHE1 proteins. We have earlier demonstrated a shift in the apparent molecular weight of the NHE1 protein with the removal of carbohydrate groups [14].

Figure 2B illustrates the wild type and mutant NHE proteins NHE-582, NHE-566 and NHE-562. The mutant proteins were expressed at less than 20% of the level of the wild type, though this must be considered only a rough estimate because the blot was exposed for a longer period of time to visualize the smaller NHE fusion proteins (resulting in the overexposure of the wild type protein). Most of the immunoreactive mutant proteins were expressed as the smaller putatively un-glycosylated species of the NHE1 protein.

Figure 2C,D show examples of, and a summary of, the surface targeting of the wild type and mutant NHE1 proteins. Wild type NHE1 and the NHE-700 and NHE-675 mutant proteins targeted approximately 80% of the plasma membrane. This decreased with the length of the C-terminal region in step with a final abrupt significant decrease from about 30% to less than 10% with a shortening of NHE1 from amino acid 566 to amino acid 562.

**Figure 3:**
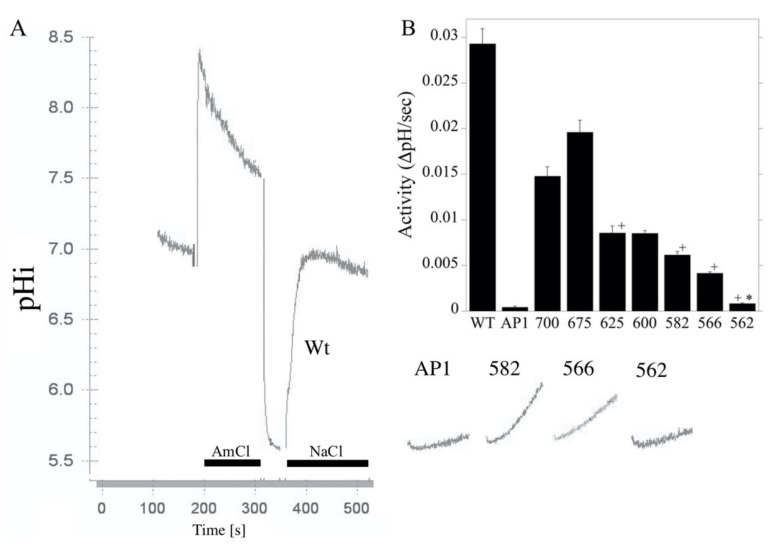
Activity of wild type NHE1 and mutants NHE-562, NHE-566, NHE-582, NHE-600, NHE-625, NHE-675 and NHE-700. (**A**) example of the activity of stably transfected cells. Acidosis was induced with NH_4_Cl (AmCl), as described in the Materials and Methods section. To induce acidosis, there was a brief Na-free treatment between AmCl and NaCl. NaCl indicates the recovery period from acidosis in a medium that contains NaCl. For clarity, only the recovery period is shown from the NHE1 mutant proteins. (**B**) is a summary of the raw activity results (mean ± S.E., *n* > 6). All values were significantly reduced from wild type levels at *p* < 0.01. + indicates values that were significantly different from the next largest expressed protein at *p* < 0.01. * indicates values that were significantly different from AP1 cells at *p* < 0.05.

The NHE activity of the mutants is shown in Figure 3. The NHE-700 and NHE-675 mutant proteins possessed reasonable activity, about half that of the wild type. This dropped by about half again in the NHE-625 and NHE-600 mutant proteins. The NHE-582 and NHE-566 decreased somewhat more, and there was an abrupt drop in activity in the NHE-562 mutant compared with the NHE-566 mutant that was just four amino acids larger. The correction of the activity of the mutants for the levels of surface processing or expression levels resulted in relative activities of the mutant proteins that were greater than those of the control. This suggested that changes in expression and targeting mediate the reduced NHE activity. It should be noted however, as stated above, the low levels of expression and targeting make these corrections inaccurate, and the data can only be interpreted as general support for the hypothesis that reduced activity is due to decreased expression and targeting—not due to a defect in the NHE1 transport mechanism itself.

### 2.2. Characterization of Amino Acids 563–566 of the Cytosolic C-Terminal Tail

**Figure 4:**
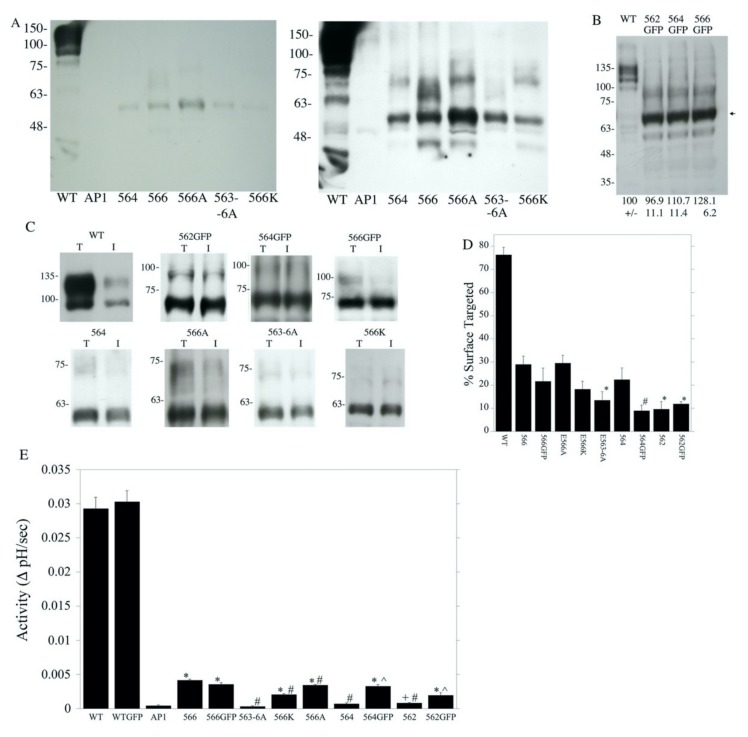
Effect of alterations of amino acids 563–566 on NHE1 expression and targeting. (**A**) Western blot of stable cell lines containing the NHE1, wild type (WT), NHE-564, NHE-566, NHE-E566A, NHE1-E563-6A, and NHE-566K proteins. The left panel shows a shorter exposure of film. The right panel shows a longer exposure of film. Both Western blots were probed with anti-HA antibodies. (**B**) Western blot of cell lines that expressed the following proteins: NHE1 fused to GFP, WT, full length NHE1 fused to GFP, NHE-562-GFP, NHE-564-GFP and NHE-566-GFP. Numbers indicate expression levels relative to that of full-length the NHE1 protein fused to GFP, *n* = 3. The Western blot was probed with anti GFP antibodies. Arrow indicates the principle immunoreactive, shortened protein NHE1 fused to GFP: NHE-562-GFP, NHE-564-GFP, and NHE-566-GFP. (**C**) Surface localization of wild type (WT) and mutant NHE1 proteins in AP1 cells. The results are shown as mean ± the S.E., *n* = at least 3 determinations. Total cell lysates (T) and unbound intracellular lysates (I) were examined by Western blotting with an anti-HA antibody to identify the present NHE1 protein. WT and mutant cell lines are indicated in (**C**). (**D**) summary of results of surface processing. All values were significantly reduced from wild type levels at *p* < 0.01. # and * significantly different from NHE-566 at *p* < 0.01 and *p* < 0.05, respectively. (**E**) Effect of alterations of amino acids 563–566 on NHE1 activity. Results are shown as mean ± S.E., *n* > 6. All values (except WTGFP) were significantly reduced from wild type levels at *p* < 0.01. * and + significantly different AP1 cells *p* < 0.01 or *p* < 0.05, respectively. ^, significantly greater than same length construct without the added GFP protein at *p* < 0.01. #, significantly different from NHE-566 at *p* < 0.01.

Because the deletion of four amino acids 563–566 (amino acids IAGE) caused notable changes in both surface targeting and activity, we examined this region in detail for the role of the particular amino acids that were present. A number of other mutations were made: NHE-564, NHE-E566A (amino acid 566 changed to Ala and followed with a hemagglutinin (HA) tag), NHE-563-6A (amino acids 563 to 566 changed to Ala and followed with an HA tag), NHE-E566K (changing amino acid 566 to Lys and followed with an HA tag), NHE-562-GFP, NHE-564-GFP, and NHE-566-GFP (which end at the indicated amino acid and are followed by a green fluorescent protein (GFP) tag, Figure 1A). The expression of these mutants was much lower than that of the wild type protein. Though the low level of protein that was present made only rough quantification possible, this was roughly 10% or less of the wild type for all the proteins except for NHE-E566A, which was less than 15% (Figure 4A). The main immunoreactive protein was about 60 kDa. The calculated molecular weights of the proteins were all about 67 kDa (Table 1), which is in reasonable agreement with the sizes shown. Membrane proteins often have slightly anomalous apparent molecular weights in SDS-PAGE. A longer exposure of the film (right panel) shows that some higher molecular weight immunoreactive proteins were also present in the different cell lines, which may have been caused by the NHE1 protein aggregating with other proteins. The level of expression of the shortened NHE1 protein, which was fused to GFP, was approximately equivalent to that of the wild type NHE1 protein (Figure 4B).

We examined the targeting of this group of mutants (Figure 4C,D). All were less efficiently targeted to the cell surface than the wild type. The addition of GFP to the NHE proteins that were shortened by two or four amino acids did not improve surface targeting relative to NHE-566. Changing amino acid 566 to Lys did not improve targeting. Changing all amino acids 563–566 to Ala decreased targeting relative to NHE-566. Changing only amino acid 566 to Ala made no change in targeting compared to NHE-566 without the mutation (Figure 4D).

An examination of the activity of this second group of mutants is shown in Figure 4E. The wild type NHE1 protein with the HA tag and WT NHE1 fused to GFP had activity that was much greater than that of the shortened mutants. However, the addition of GFP to the wild type NHE1 protein did not enhance activity. When comparing NHE1 shortened at amino acids 562 or 564, changing the tag from HA to GFP increased activity. This did not occur with amino acid 566. Changing amino acids 563–566 to Ala decreased activity, while mutating amino acid 566 to either Lys or Ala did not improve activity.

**Figure 5:**
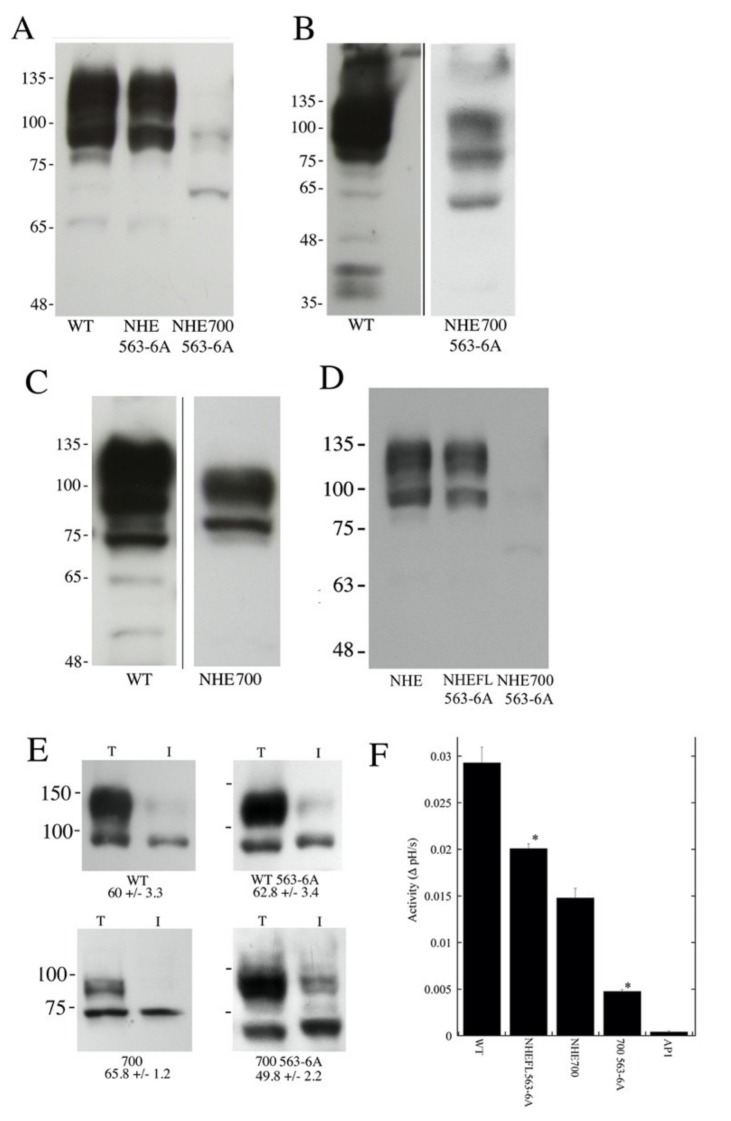
Effect of mutation of amino acids 563–566 on NHE1 with extended carboxyl terminal tails. WT, wild type NHE1, NHE-563-6A (full length wild type NHE1 with amino acids 563–566 changed to Ala), NHE-700 (NHE1 shortened and terminated at amino acid 700), NHE1 shortened to amino acid 700, 700 563-6A, NHE1 shortened to amino acid 700 and with amino acids 563–566 changed to Ala, AP1, and cells with no Na^+^/H^+^ exchanger. (**A**) Western blot of cell lysates from wild type (WT) NHE1-containing cells, wild type full-length NHE1 with amino acids 563–566 changed to Ala (NHE FL-563-6A), and NHE1 shortened to amino acid 700 and with amino acids 563–566 changed to Ala (NHE-700-563-6A). (**B**) Long exposure of Western blot of NHE1 from wild type (WT)-containing cells and cells containing NHE-700-563-6A. (**C**) Long exposure of the Western blot of NHE1 from wild type (WT)-containing cells and cells containing NHE-700. (**D**) Comparison of levels of NHE1 and NHE1 with the mutation of 563–566 to Ala. Equal amounts of cell lysates that contained each type of NHE1 were loaded onto gels and immunoblotted with anti-HA antibodies. (**E**) Surface processing of wild type (WT) NHE1-containing cells, NHE1 with amino acids 563–566 changed to Ala (NHE-FL-563-6A), as well as NHE1 shortened to amino acid 700 along with amino acids 563–566 changed to Ala (700 563-6A). Numbers indicate the percent of proteins that were targeted to the cell surface. Results are the mean ± S.E. of at least 3 experiments. (**F**) Activity of wild type NHE1 and mutants. Cells that contained wild type or mutant proteins were acidified with ammonium chloride, as described in the Materials and Methods sections. The initial rate of recovery was measured and recorded as ΔpH/s. *n* > 8. * *p* < 0.001 compared with equivalent length protein without 563–566 to Ala mutation.

To determine if the mutation of the amino acids 563–566 could affect NHE1 protein expression and activity, we mutated these residues to Ala in the full-length protein and the NHE1 protein that terminated at amino acid 700. We then examined the expression, targeting and activity of the NHE1 protein. Figure 5A–C show the expression levels of the proteins. Figure 5A shows an immuno-blot that was probed with anti-NHE1 tag (HA) against equal amounts of cell lysate wild type NHE1, wild type NHE1 with the mutation of 563–566 to Ala, and NHE1 truncated at amino acid 700 along with the mutation of amino acids 563–566 to Ala. The larger full length NHE1 proteins were quite visible, though the truncated mutant was much less visible. To confirm the presence of the truncated protein, we exposed the blot for a longer period of time (Figure 5B), and the smaller NHE protein was more apparent. Figure 5C shows a similar long exposure that illustrates wild type NHE1 and NHE-700 (without the 563-6A mutation).

To quantify the relative levels of NHE1 and NHE-700 with the mutation of 563–566 to Ala, we repeated the Western blotting (Figure 5D). Figure 2 shows the quantification of the NHE-700 expression, which was approximately 27% of the wild type NHE1 and thus significantly greater than that of NHE-700-563-6A, at *p* < 0.01. The introduction of the 563-6A mutation into the full-length wild type NHE1 protein caused an approximately 20% decrease in expression levels (Figure 5D) to 81 ± 2.3% of the controls, though this was not statistically significant. The NHE 700-563-6A protein was significantly reduced to 6.4% of the wild type level (6.4% ± 2.6). The measurement of surface processing (Figure 5E) showed that the introduction of the 563–566 mutation did not affect surface processing in the wild type protein or NHE-700, but the surface processing of the mutant that ended at amino acid 700 was slightly (but not significantly) reduced (Figure 5E). The measurement of NHE1 activity is shown in Figure 5F. The introduction of the mutation significantly reduced the activity of the protein in the wild type protein by about one third. In the NHE1 protein that was truncated at amino acid 700, the effect was greater, reducing activity by about two thirds.

### 2.3. Effects of Mutations of Amino Acids 563–566 on Longer NHE1 Proteins

**Figure 6:**
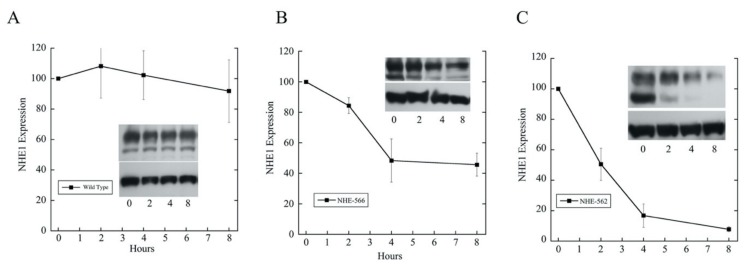
Protein levels of wild type and mutant NHE1 proteins were measured at time 0, (starting time) and up to 8 h after cycloheximide (50 μM) treatment. Equal amounts of total proteins were loaded in each lane, and NHE1 levels were then determined by Western blotting against the HA tag on NHE1. Quantification was estimated by using the Image J 1.35 software. Insets are example Western blots that show NHE1 levels in the upper panel in comparison to tubulin levels (lower panels). Time points are at 0, 2, 4 and 8 h. Results are mean ± S.E. of at least 3 experiments.

Previously, we showed that the large scale deletion of the NHE1 C-terminal tail resulted in a more rapid degradation of the mutant proteins compared to the wild type [13], but we only made a rough localization of the responsible general regions. Because we knew that protein degradation could be involved, we examined the effect of the deletion of amino acids 563–566 by comparing the degradation rates of the wild type NHE1 protein with that of the NHE-566 and NHE-562 proteins (Figure 6). The wild type NHE1 protein was relatively stable with only a minor decline in the level of the protein over eight hours. In contrast, both the NHE-566 and NHE-562 proteins were much more unstable. The NHE-566 protein was reduced by about 50% in eight hours. The NHE-562 protein was decreased by over 90% over eight hours. The degradation of the NHE-562 protein had a half-life of approximately 1.5 h (fit to an exponential decay *r* = 0.99, not shown). After two hours, the wild type protein had not declined appreciably, while the NHE-566 protein declined by approximately 17%.

### 2.4. Examination of Amino Acids 562–568 Binding to the C-Terminal Tail Region

**Figure 7:**
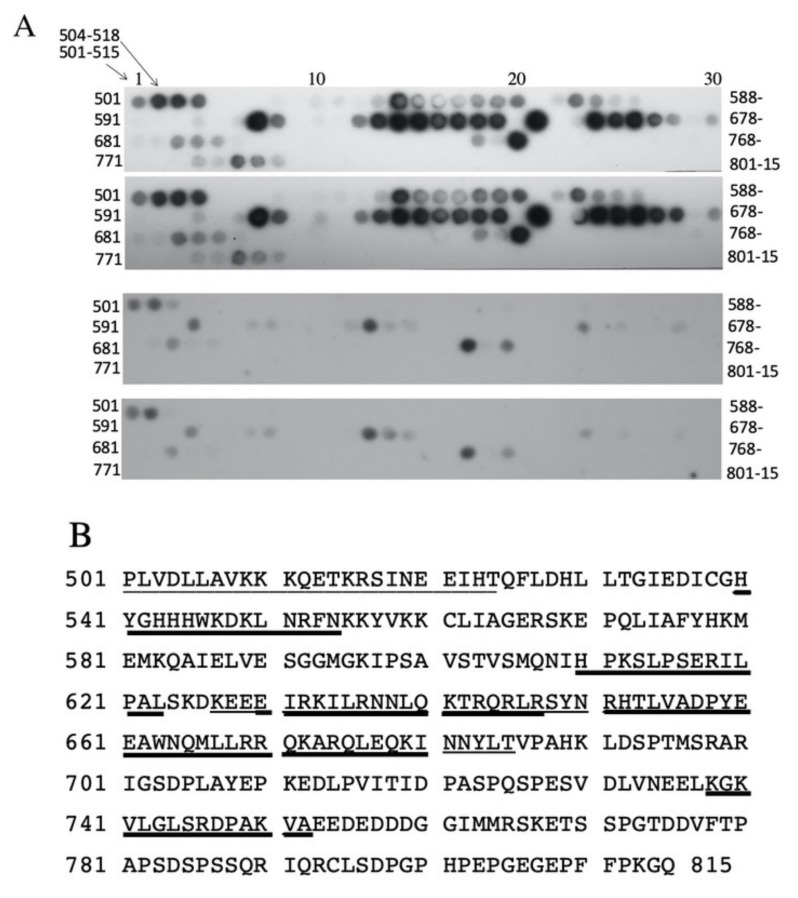
Synthetic peptide array blot [15] of amino acids 501–815 of the human NHE1 C-terminus region that was probed with HA-tagged peptide-containing amino acids (^562^LIAGERS^568^). The N-terminal peptide of the peptide array is indicated. Each spot on the array contained a peptide of 15 amino acids in length. The adjacent peptide was shifted by three amino acids, as indicated. (**A**) Illustration of results of probing (in duplicate). The top two panels were probed with the peptide of the N-GL**IAGE**RSYPYDVPDYAG sequence. The bottom two panels were probed with the peptide N-GLAAAARSYPYDVPDYAG sequence. (**B**) NHE1 C-terminal amino acids indicating regions of stronger binding to the tagged peptide. Underlined sequences indicate the amino acids of the 15mer peptide(s) that bound to the probe, and the thicker line indicates a higher level of binding. Results are typical of four experiments.

To examine if the amino acids of the ^562^LIAGERS^568^ of NHE1 region could bind to other parts of the NHE1 carboxyl terminal tail, we made a synthetic peptide of this region with a tag for detection. We then used it to probe an oligopeptide array of amino acids 501–815 of the human NHE1 C-terminus (Figure 7). The experimental peptide was compared to a control peptide that was changed to have four consecutive Ala residues at amino acids 563–566. There were several discrete regions where the experimental peptide bound to the peptide array, much more so than the control peptide. The binding regions are summarized in Figure 7B and Supplementary Figure S1A–C. The regions of stronger, more apparent binding that was much greater than control were contained between amino acids 540–554, 609–623, 630–647, 651–665, 660–681, and 738–752, though some peptides within these general regions did not bind the probe. A region from 501–524 bound to the experimental peptide, but the control peptide also bound to this region. We compared the regions of the C-terminal tail that bound to the ^562^LIAGERS^568^ sequence with their hydrophobicity. Supplementary Figure S2 shows a plot of the binding of the peptide versus its hydrophobicity. There was no correlation between hydrophobicity and the peaks of the ^562^LIAGERS^568^ sequence that bound to the NHE1 C-terminal tail. Some binding was in more hydrophilic regions, and some was in more hydrophobic regions that also contained troughs in the level of binding. The entire C-terminal region was, in fact, not very hydrophobic.

### 2.5. Multiple Sequence Alignment of NHE1

**Figure 8:**
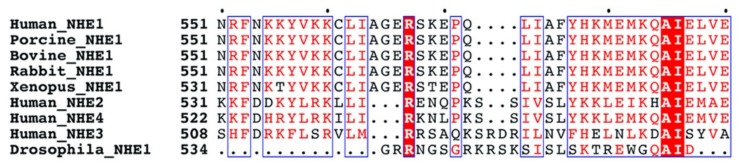
Multiple sequence alignment of amino acids 551–590 of the human NHE1 (P19634) with porcine NHE1 (NP001007104), bovine NHE1 (Q28036), rabbit NHE1 (P23791), *Xenopus* NHE1 (NP_001081553.1), human–NHE2 (NP_003039.2), human–NHE3 (NP_004165.2), human–NHE4 (NP_001011552.2), and *Drosophila*–NHE1 (NP_608491.3) proteins. Amino acids in the red box show conserved residues. Red colored amino acids are similar in type. Red shading indicates conserved amino acids throughout all species examined. The alignment was made manually.

Figure 8 illustrates a multiple sequence alignment of amino acids 551–590 of the human NHE1 protein with several other species. The ^562^LIAGERS^568^ sequence was completely conserved in the vertebrate NHE1 isoform of the Na^+^/H^+^ exchangers. However, it was not conserved in isoforms 2–4 of human NHEs, nor was it conserved in the *Drosophila* NHE1 protein.

## 3. Discussion

We earlier [13] demonstrated that stop codon polymorphisms in the human mammalian NHE1 protein resulted in reductions in the level of expression and activity of the protein. Mutant NHE1 proteins that were truncated at amino acids 321, 449 and 543 lost activity and did not target to the plasma membrane. Additionally, the protein for these mutants was more rapidly degraded than the wild type. However, a longer version of the NHE1 protein, terminating at amino acid 735, targeted to the cell membrane with a reasonably high activity despite the loss of 80 amino acids. This study showed that mutations that were equivalent to human polymorphisms had very significant effects on function. However, the precise location of elements on the protein that caused defective expression, trafficking, and activity remained unknown and would be of interest in understanding effects of other, as yet uncharacterized human polymorphisms.

Several types of evidence suggest that amino acids 563–566 have a specific effect on the NHE1 protein and a specific function. It was only when the protein was shortened past amino acid 566 that most of the activity was eliminated. The NHE-582 and NHE-566 proteins still retained significant Na^+^/H^+^ exchanger activity, but we found that the deletion of four more amino acids to make NHE-562 abolished most of the activity of the protein (Figure 3). The effect of deletion was not a general effect on the length of the protein. Firstly, the effect occurred when the GFP protein was added to the C-terminal of NHE1. The addition of GFP only had a very minor effect on restoring activity and targeting. Secondly, we changed the sequence of amino acids 563–566 so they were Ala in versions of NHE1 with longer tails (Figure 4). This mutation significantly reduced the activity of the protein in the wild type protein by about one third, while the effect was greater in the NHE1 protein that truncated at amino acid 700, reducing activity by about two thirds. Part of the effect was likely due to reduction in expression levels and targeting, especially in the protein that ended at amino acid 700.

The cause of reduced levels of the protein with the removal of amino acids 563–566 was likely due to an increase rate of the degradation of the protein. We showed earlier that large truncations of the NHE1 caused the enhanced degradation of the protein [13]. Here, (Figure 6), we demonstrated that removal of these four amino acids caused much of this effect. The protein that was truncated at amino acid 562 was degraded much more quickly than the protein that was truncated at amino acid 566. We noticed that the smaller molecular weight form of the NHE1 protein, which was immature and unglycosylated [14], appeared to more rapidly degrade than the full-size glycosylated protein in the shortened protein. Mutant NHE1 proteins have been shown to sometimes target preferentially to the endoplasmic reticulum [16]. The endoplasmic reticulum (ER) can recognize abnormal protein conformations, and heart shock protein 70 may be involved in processing of NHE1 to the ER because these proteins are known to interact with each other [16,17]. ER-associated degradation directs the degradation of a variety of misfolded and normal proteins. It may be that the smaller molecular weight forms of the NHE1 proteins are more susceptible to mistargeting and ER-associated degradation. We did find enhanced degradation rates and decreased targeting to the cell surface of the shorter NHE1 proteins. We did not document a changed in the degradation rates of the full length NHE1 with the 563-6A mutation; however, the levels of the protein were reduced from the wild type NHE1 and the targeting of the mutant was unaffected. Future experiments may explore whether this sequence causes ER-associated degradation in full length NHE1 or in other proteins.

What could be other mechanisms of the decreased activity, targeting and greatly enhanced degradation shown in our shortened NHE1 proteins? We examined putative intramolecular interactions between amino acids ^562^LIAGERS^568^ and other regions of the NHE1 C-terminus (Figure 8). The sequence bound to several other regions of the tail. Which of these occurs in vivo is not yet clear. However, it is apparent that this region could be involved in intramolecular interactions with the C-terminus of NHE1. We hypothesize that these stabilize the tail region of the protein, possibly leading to enhanced folding, which leads to greater the correct targeting and decreased degradation of the protein. With loss of these regions, the stability of the protein may be decreased, leading to misfolding and enhanced ER-associated degradation.

The LIAGER sequence motif and flanking amino acids are highly conserved among Na^+^/H^+^ exchangers (Figure 8), at least in the vertebrate species. Other isoforms of the protein do not have this sequence, nor does invertebrate NHE1 of *Drosophila*. A number of regulatory features of Na^+^/H^+^ exchangers appear to be conserved among the isoforms, but this seems to vary when comparing one isoform to another [2,18]. Running the LIAGER or LIAGERS sequences in the eukaryotic linear motif (ELM) database for functional sites in proteins [19] yielded only a low probability N-terminal motif (^M{0,1}[FYLIW][^P) that initiates protein degradation. This only matched the amino acids “LI,” and was not a good match in either function, the location on the protein, or to the full sequence. The LIAGER sequence therefore appears to be previously undescribed.

Calmodulin binds to two sites on the NHE1 C-terminal tail. One, a high affinity binding site at amino acids 636–656, modulates activity and serves as an autoinhibitory domain [20]. It was interesting that the ^562^LIAGERS^568^ sequence bound to peptides of this region in relative larger amounts. The significance of this is not yet clear at this time.

The LIAGER sequence is contained within or near several other loosely described motifs of NHE1. The amino acids 562–580 have been suggested to be involved in the dimerization of the NHE1 protein, though specific amino acids within this region were not analyzed [21]. Additionally, it is interesting to notice that the ^562^LIAGERS^568^-containing peptide did not associate with itself in our peptide screen, though this does not preclude it being involved in dimerization through other parts of the C-terminal of NHE1. Amino acids in this general region (542–598) have also been suggested to bind ATP, though ATP hydrolysis is not required for NHE1 activity [22]. Precise amino acids within this region binding ATP have not yet been elucidated. It is also worth note that there are proximal PI(4,5)P2 binding sites that overlap with binding sites for the cytoskeletal linker proteins ezrin, radixin, and moesin (ERM proteins) (reviewed in [18]). The ERM binding sites are located close to amino acids 508–512 and 551–560 [2,23], while the PI(4,5)P2 binding sites are reported at amino acids 513–520 and 556–564 (rat) of NHE1 (equivalent to 509–516 and 552–560, respectively, of human NHE1) [2,24]. These sites are close to the “LIAGERS” sequence.

In summary, we suggest that the ^562^LIAGERS^568^ sequence defines a new sequence that is important in the maintenance of NHE1 protein levels and activity. As the NHE1 tail has been demonstrated to be mutated and shortened in some humans [13], it will be important in future studies to examine other polymorphisms and determine if they account for human pathologies, as has recently been demonstrated for the membrane domain [11].

## 4. Materials and Methods

### 4.1. Materials

Surface labeling was done with Sulfo-NHS-SS-Biotin, which purchased from Thermo Fisher (Waltham, MA, USA). Synthetic oligonucleotides for site-specific mutagenesis were from IDT (Coraiveille, IA, USA). For pH_i_ measurements, 2′,7-bis(2-carboxyethyl)-5(6) carboxyfluorescein acetoxymethyl ester (BCECF-AM) was purchased from Molecular Probes, Inc. (Eugene, OR, USA). For transfection, the Lipofectamine^TM^ 2000 reagent was purchased from Invitrogen Life Technologies (Carlsbad, CA, USA). For protein stability studies, cycloheximide was purchased from Santa Cruz Biotechnology Inc, (Dallas, TX, USA). Fisher Scientific, BDH (Toronto, ON, Canada) or Sigma-Aldrich (St. Louis, MO, USA) supplied other chemicals of analytical grade. The plasmid pYN4+ has an HA (hemagglutinin) tag, as described earlier [25], and was used to express cDNA for the human NHE1 protein. The plasmid pmEmerald-dectin-1A-N10 was a generous gift from Dr. N. Touret, of the Department of Biochemistry, University of Alberta. The polyclonal anti-GFP antibody was a generous gift of Dr. Luc Berthiaume, Department of Cell Biology, University of Alberta.

### 4.2. Cell Culture and Stable Transfection

To characterize the activity of the wild type vs. mutant Na^+^/H^+^ exchangers, we used a cell line that was deficient in endogenous NHE1 AP1 cells. This mutant cell line is derived from Chinese hamster ovarian cells [26]. AP1 cells were stably transfected with the Lipofectamine™ 2000 reagent. The pYN4+ plasmid and mutants contained a neomycin resistance cassette that allowed for selection of stably transfected cells with the G418 antibiotic. Whenever necessary, cell lines were re-established from frozen stocks between passage numbers 5 and 11. At least two stable cell lines were made independently of each mutant [26].

### 4.3. Mutant Plasmid Construction

The plasmid pYN4+ has the full-length cDNA of human NHE1, followed by an HA tag. To create shortened NHE1 sequences followed by an HA tag, we inserted a XhoI site before the tag through site directed mutagenesis by using these primers (F-GAGGGAGAACCGTTCTTCCCCAAGctcgAG gatgacGGCCGCATCTTTTACCC R-GGGTAAAAGATGCGGCCgtcatcCTcgagCTTGGGGAAGAAC GGTTCTCCCTC), as described earlier [13]. Another mutagenesis was used to insert an upstream XhoI site in the appropriate reading frame. This inserted second XhoI site was designed so the intervening XhoI–XhoI piece could be deleted by restriction enzyme digestion. After the deletion, the vector was re-ligated, thus giving the appropriate shortened plasmid at the indicated amino acid of NHE1 and in frame with the HA tag. The primers that were used for mutations are listed in Table 1. DNA sequencing confirmed the fidelity of the mutations and the DNA amplification. In a series of progressive mutations, NHE1 proteins were shortened at amino acids 700, 675, 625, 600, 582, 566, 564, and 562, and they were fused to the HA tag. Constructs are designated in the format NHE-700 as appropriate (Table 1, Figure 1). Additional mutations altered amino acid 566 to Ala, or Lys (NHE-566A and NHE -566K, respectively). The mutant NHE-563-6A had the last four amino acids of NHE1 changed to Ala (and was also fused to HA in frame, as described above).

The plasmid pmEmerald-dectin-1A-N10 contained dectin followed by GFP. The enzymes Nhe1 and AgeI were used to remove dectin, and the resultant vector fragment was used for cloning. The primers 5′NHEprimer (AAGAATTCgctagcGCCACCATGGTTCTGCGGTCTGGCATC) and 3′NHEPrimer (5′-CCGAATTCcccgggGGAACGTCATATGGATAGGATCCTGC-3′) were used to amplify NHE1 from the plasmid pYN4+ and to add Nhe1 and Xma1 sites for cloning into the pmEmerald plasmid fragment. This resulted in an expression vector with an in frame GFP that was fused to the entire NHE1 C-terminal. To create shortened versions of the NHE1 protein that was fused to GFP, a strategy that similar to that described above was used. A primer set based on the (NHE1GFP + Xhof, CCGGACTATGCAGGATCCTATctcgagCCCCCGGTCGCCACCATGGTGAG) sequence was used to introduce an XhoI site into the NHE1-GFP-containing vector by site-specific mutagenesis. The resultant plasmid (pNHE1GFP + Xho) was used with the primers that was indicated in Table 1 to make NHE1 that was fused to GFP with a C-terminal tail that was shortened at amino acids 562, 564 and 566 (Table 1, Figure 1). The resultant constructs terminated at the indicated amino acid, followed by an HA tag and GFP.

### 4.4. Cell Surface Expression

Cell surface proteins were labeled with Sulfo-NHS-SS-Biotin to examine the targeting of the NHE1 protein [27]. Total cell proteins were solubilized and the cell surface proteins, including the NHE1, were removed by using immobilized streptavidin resin. We then immunoblotted for HA or GFP-tagged NHE1 protein thereby examining the removal of cell surface proteins with streptavidin-agarose [28,29]. The levels of the immunoreactive protein present were estimated by using Image J 1.35 software (National Institutes of Health, Bethesda, MD, USA). Quantification was of both the upper and lower HA-immunoreactive-specific immunoreactive bands of the NHE1 protein where used, and the percentage of the protein targeting on the cell membrane was calculated by using the equation:

(1) $$\frac{\mathrm{Total}-\;\mathrm{Unbound}}{\mathrm{Total}}\times 100\%$$

### 4.5. SDS-PAGE and Immunoblotting

We examined NHE1 levels in stably transformed AP1 cell lines via immunoblotting against the GFP or HA tag of the NHE1 protein, where appropriate [30]. Cell lysate proteins were separated on SDS-PAGE (10%) gels and transferred to nitrocellulose. One hundred μg of protein of control or experimental lysates were run in triplicate for quantification. The BioRad D/C^TM^Protein Assay kit measured protein concentrations. The primary antibody to identify tagged NHE1 was anti-HA monoclonal antibody or polyclonal anti-GFP antibody. The secondary antibody was peroxidase-conjugated goat anti-mouse antibody or anti-rabbit antibody (Bio-Can, Mississauga, Ont., Canada). Protein reactivity and quantification was performed as above.

### 4.6. Protein Degradation of Wild Type and Mutant NHE1 Proteins

To examine NHE1 protein stability, stable cells lines of wild type and mutant NHE1-containing cells were treated with 50 μM cycloheximide for up to 8 h. Cell lysates were examined for NHE1 protein expression by Western blotting versus the HA tag on the NHE1 protein. The quantification of NHE1 protein levels was done by using the Image J 1.35 software, as described above. The decay of the NHE-562 protein was fit on an exponential decay curve by using the program Kaleidagraph 4.5 (Synergy Software).

### 4.7. Peptide Blot

The peptide constructs of amino acids 501–815 of the human NHE1 C-terminus were made as described earlier [15]. Peptides of a length of 15 amino acids were made and shifted by three amino acids. They were synthesized on acid-hardened cellulose membranes and derivatized with a polyethylene glycol spacer. To probe the peptide array, a synthetic peptide was made of the N-GLIAGERSYPYDVPDYAG-C sequence, which contained the ^562^LIAGERS^568^ amino acids of NHE1 followed by a hemagglutinin (HA) tag (underlined) and with both N and C terminuses flanked by a Gly residue. A control peptide had the central part of the key sequence changed so that it had four consecutive Ala residues N-GLAAAARSYPYDVPDYAG-C. To probe the array, membranes were activated with MeOH for 1 min and then washed twice with H_2_O for 1 min each time. The membrane was equilibrated for 2 h with a binding buffer (50 mM Tris-HCl pH 7.5, 150 mM NaCl, 0.1% Triton X 100) and blocked for 1 h with a binding buffer that contained 1 µM bovine serum albumin at room temperature. The membrane was then incubated with a binding buffer that contained 1 μM synthetic peptide at 4 °C overnight. It was washed three times with a binding buffer for 10 min each and then incubated with antibody against the HA tag. Immunodetection was performed with enhanced chemiluminescence, as described earlier [31]. The quantification of the peptide blot was carried out by using Image J software (National Institutes of Health). The hydrophobicity of the amino acids 501–815 of NHE1 was determined by using the ExPASy Protein server’s Kyte and Doolittle analysis with a window size of 15 [32].

### 4.8. Intracellular pH Measurement

To characterize pH_i_ and NHE1 activity, cells were grown to approximately 80–90% confluence on glass coverslips. BCECF was loaded into cells, and fluorescence was measured by using a Photon Technologies International (London, Ont., Canada) Deltascan spectrofluorometer. NHE1 activity was measured after an acute acid load was induced. Ammonium chloride (50 mM × 3 min) addition was followed by removal to induce the acute acidosis. ΔpH/s during the first 20 s of recovery in an NaCl-containing medium was measured. The rate of recovery in the wild type NHE1 protein was compared with the rate of recovery in the shortened NHE1 mutants. A calibration of pH_i_ fluorescence was performed for every sample by using nigericin [26]. Results are the mean ± S.E. of at least seven experiments. Statistical significance was determined by using the Wilcoxon–Mann Whitney test.

## Figures and Tables

**Table 1:** Oligonucleotides that were used for site specific mutagenesis. Lowercase indicates altered bases. Only the forward primer is shown.

Product Name	Start Plasmid	Synthetic Oligonucleotide	Description	Calculated Molecular Wt
Wild type	A (Wt NHE HA tag)	N/A	815-HA	95220
Wild type	B (WT NHE1 GFP tag)	N/A	815-GFP	121612
NHE-700	A	5′-CCATGTCTCGGGCCCtCgagGGCTCAGACCCACTG-3′	Stop @AA 700-HA	81187
NHE-675	A	5′-GGCAGAAGGCCCGGCtcgaGGAGCAGAAGATCAAC-3′	Stop @AA 675-HA	80229
NHE-625	A	5′-CTGCCAGCACTGTCCctcGAgAAGGAGGAGGAGATC-3′	Stop @AA 625-HA	73953
NHE-600	A	5′-GGCAAGATCCCCTCTctCGagTCCACCGTCTCCATG CAG-3′	Stop @AA 600-HA	71295
NHE-582	A	5′-CCACAAGATGGAGATGctcgAGGCCATCGAGCTGGTG-3′	Stop @AA 582-HA	69569
NHE-566	A	5′-GTGTCTGATAGCTGGCGAGCtCgagAAGGAGCCCCAGC TCATTG-3′	Stop @AA 566-HA	67579
NHE-564	A	5′-GTGAAGAAGTGTCTGATAGCTctcGAGCGCTCCAAG GAGCCCCAGCTC-3′	Stop @AA 564-HA	67393
NHE-562	A	5′-GAAATATGTGAAGAAGTGTCTcgagGCTGGCGAGC GCTCCAAG-3′	Stop @AA 562-HA	67096
NHE-E566A	A	5′-GTGTCTGATAGCTGGCgcGctggAGgatgacGGCCGCATCT TTTACCC-3′	NHE-566 with last AA as A-HA	67521
NHE-E566K	A	5′-GTGTCTGATAGCTGGCaAGctcgAGgatgacGGCCGCATC TTTTACCC-3′	NHE-566 with last 4 AA as K-HA	67578
NHE-563-6A	A	5′-ATATGTGAAGAAGTGTCTGgcAGCTgcCgcGctggAGgatgacGGCCGCATCTTTTACC-3′	NHE-566 with last 4 AA as A-HA	67493
NHE-562-GFP	B	5′-GAAATATGTGAAGAAGTGTCTcgagGCTGGCGAGC GCTCCAAG-3′	Stop @AA 562-GFP	90038
NHE-564-GFP	B	5′-GTGAAGAAGTGTCTGATAGCTctcGAGCGCTCCAAG GAGCCCCAGCTC-3′	Stop @AA 564-GFP	90335
NHE-566-GFP	B	5′-GTGTCTGATAGCTGGCGAGCtCgagAAGGAGCCCCAGCTCATTG-3′	Stop @AA 566-GFP	90521

A, alanine; AA, amino acids; GFP-green fluorescent protein tag; HA-hemagglutinin tag.

## Supplementary Materials

Supplementary materials can be found at https://www.mdpi.com/1422-0067/21/5/1737/s1. Figure S1: Binding of synthetic peptide containing the N-GL**IAGE**RSYPYDVPDYAG (experimental) sequence or the peptide N- GLAAAARSYPYDVPDYAG (control). Quantification was using Image J. Results are the mean of at least 4 determinations. (A) Binding to the peptides of the dot blot numbers 1–33 beginning at amino acid 501. Each spot on the array contains a peptide of 15 amino acids in length. The adjacent peptide is shifted by 3 amino acids towards the C-terminal. Experimental, filled bars, Control, hatched bar. Sequences of the peptide array which bound to the probe are indicated. (B,C) as in A but with dot blot numbers 34–66 and 67–101 respectively. Figure S2: Binding of synthetic peptides (as in Figure S1) in comparison with hydropathy. Hydropathy was determined using the ExPASy Protein server, Kyte and Doolittle analysis with a window size of 15.

## Abbreviations

ER	Endoplasmic reticulum
ERM	ezrin, radixin and moesin
GFP	green fluorescent protein
NHE1	Na^+^/H^+^ exchanger isoform 1
pH_i_	intracellular pH
