# Supplementary material for: Amino Acids 563–566 of the Na+/H+ Exchanger Isoform 1 C-Terminal Cytosolic Tail Prevent Protein Degradation and Stabilize Protein Expression and Activity
Source: Int J Mol Sci. 2020 Mar 3;21(5):1737. doi: 10.3390/ijms21051737 (PMC7084640; doi:10.3390/ijms21051737)
Supplement: Supplementary file 1 [file ijms-21-01737-s001.pdf]

Supplementary Figures

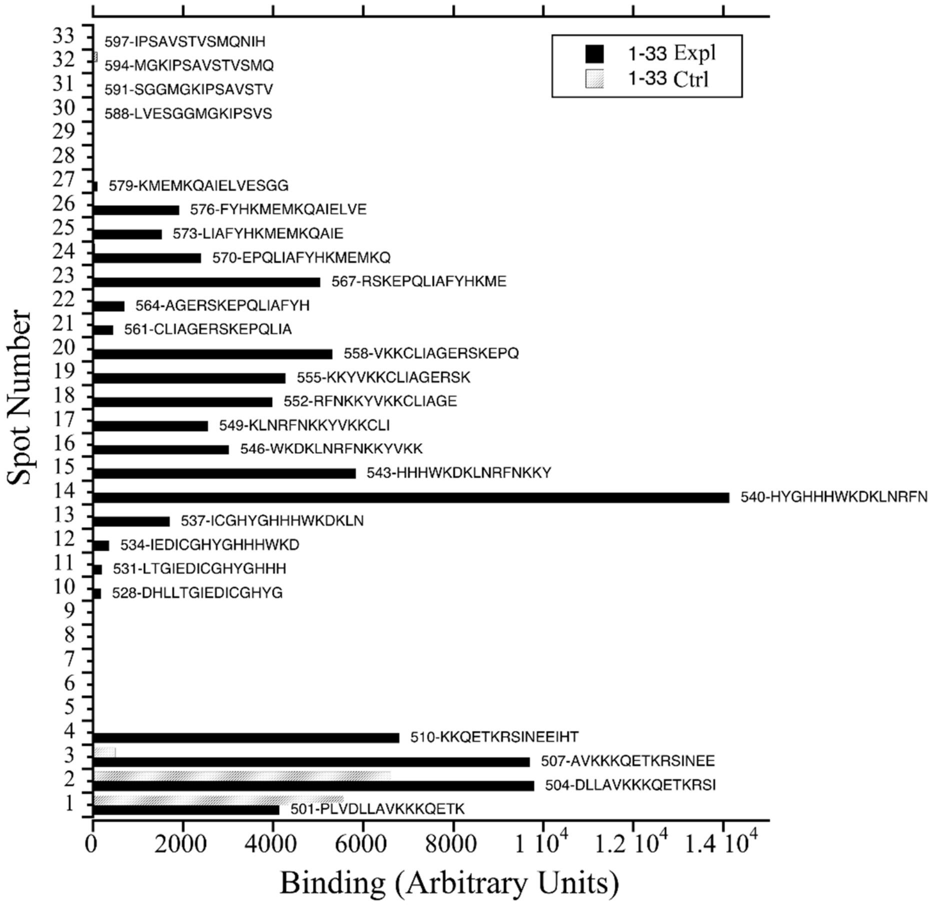

A

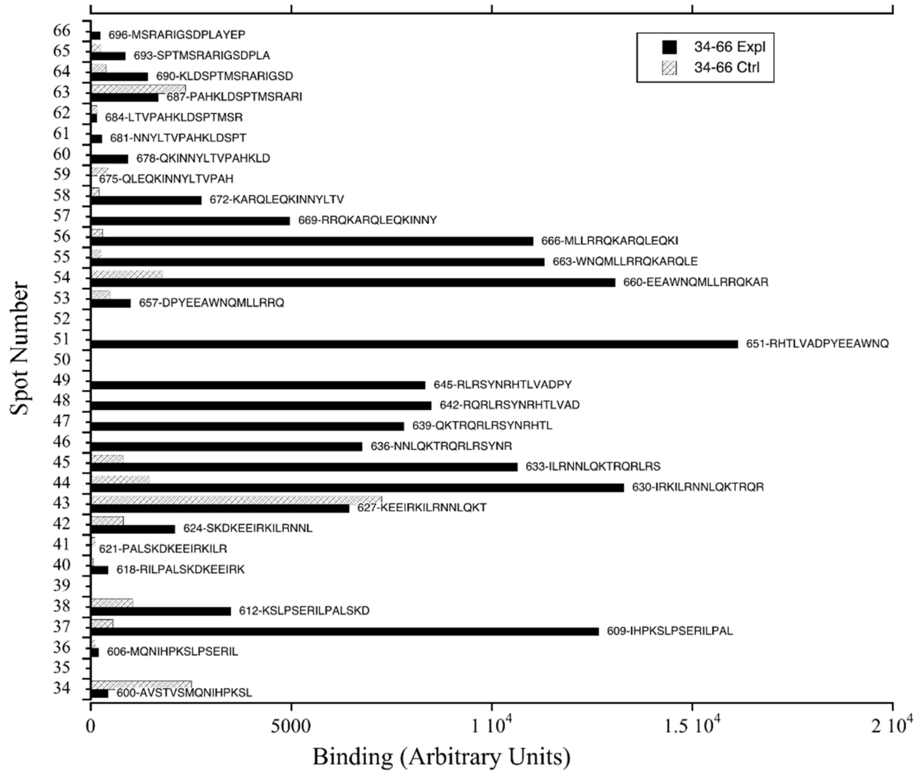

B

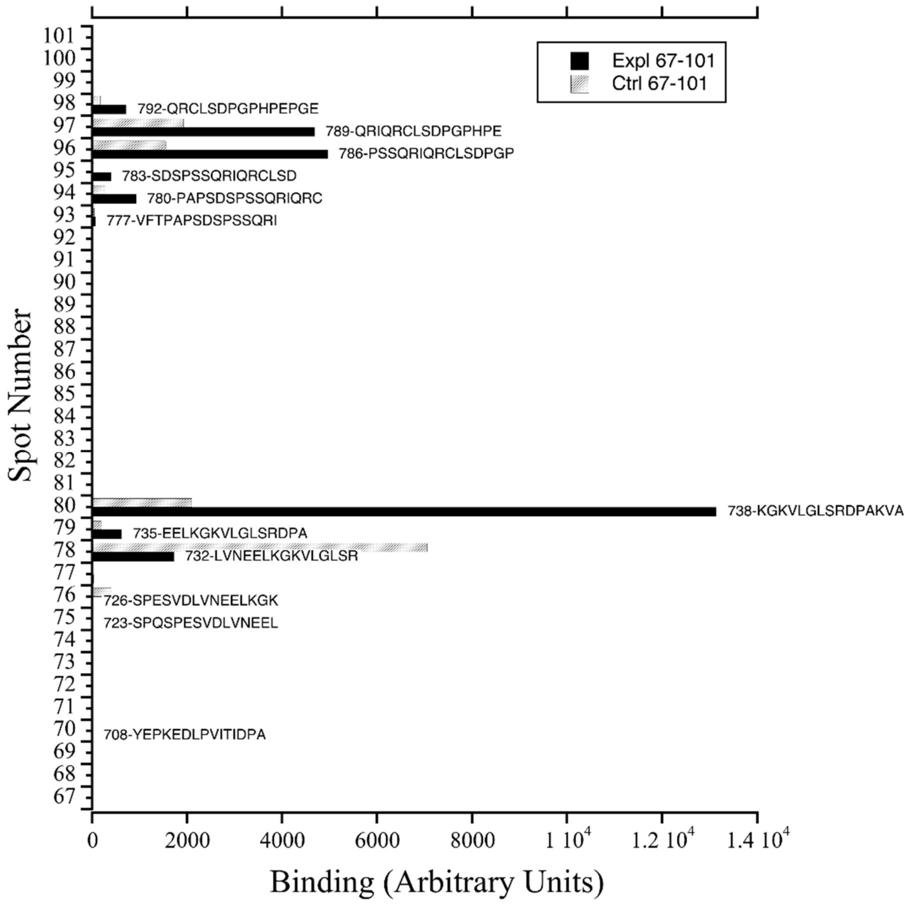

C

Figure 1. Binding of synthetic peptide containing the N-GL**IAGE**RSYPYDVPDYAG (experimental) sequence or the peptide N-GLAAAARSYPYDVPDYAG (control). Quantification was using Image J. Results are the mean of at least 4 determinations. (**A**) Binding to the peptides of the dot blot numbers 1–33 beginning at amino acid 501. Each spot on the array contains a peptide of 15 amino acids in length. The adjacent peptide is shifted by 3 amino acids towards the C-terminal. Experimental, filled bars, Control, hatched bar. Sequences of the peptide array which bound to the probe are indicated. (**B**,**C**) as in A but with dot blot numbers 34–66 and 67–101 respectively.

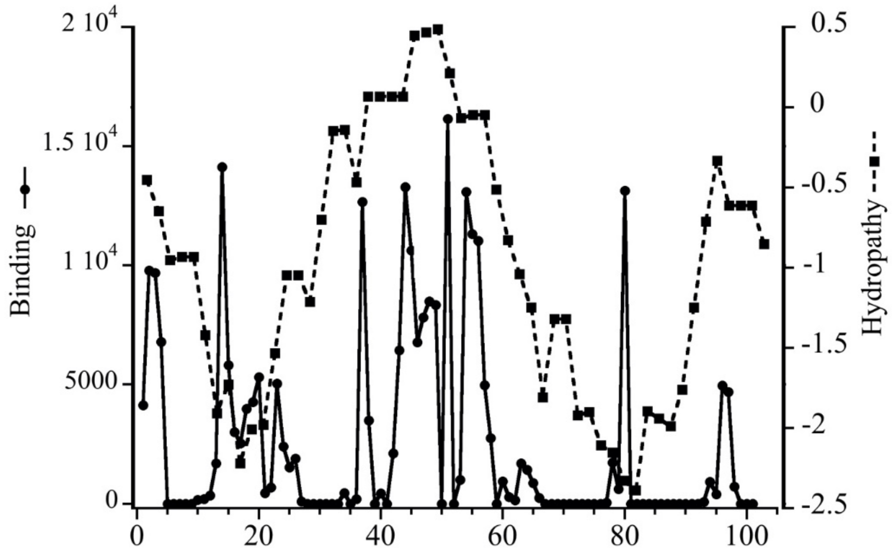

Figure 2. Binding of synthetic peptides (as in Figure S1) in comparison with hydropathy. Hydropathy was determined using the ExPASy Protein server, Kyte and Doolittle analysis with a window size of 15.
